# Neurotoxic Effects of Aromatic Organophosphate Flame Retardants Revealed by Lipidomic Analysis in Human Brain Organoids

**DOI:** 10.3390/toxics14070555

**Published:** 2026-06-25

**Authors:** Maryam Pyambri, Jordi Puigdemasa, Ana Sevilla, Joaquim Jaumot, Carmen Bedia

**Affiliations:** 1 Environmental Chemistry Department, Institute of Environmental Assessment and Water Research (IDAEA-CSIC), 08034 Barcelona, Spain; 2 Department of Analytical Chemistry, Faculty of Chemistry, University of Barcelona, 08028 Barcelona, Spain; 3 Department of Cell Biology, Physiology and Immunology, Faculty of Biology, Institute of Neuroscience, University of Barcelona, 08028 Barcelona, Spain; 4 Institute of Biomedicine of the University of Barcelona (IBUB), 08036 Barcelona, Spain

**Keywords:** organophosphate flame retardants, neural cells, reactive oxygen species, brain organoids, lipidomics

## Abstract

Organophosphate flame retardants (OPFRs) are widely used as flame-retardant additives in plastics, electronics, and building materials. However, growing evidence suggests these compounds may pose significant neurotoxic risks. This study evaluated phenotypic alterations, such as cell viability, reactive oxygen species generation, and acetylcholinesterase activity, induced by seven widely detected OPFRs in SH-SY5Y human neuroblastoma cells. Aromatic OPFRs such as triphenyl phosphate (TPhP), 2-ethylhexyldiphenyl phosphate (EHDPhP) and tricresyl phosphate (TCP) exhibited the strongest effects, including decreased cell viability, increased oxidative stress and AChE inhibition. Therefore, 3D brain organoid models were used to further explore the potential lipidomic alterations induced by aromatic OPFRs. Lipidomic analysis of brain organoids exposed to aromatic OPFRs (TPhP, EHDPhP and TCP) showed significant alterations across major lipid classes, especially glycerophospholipids, sphingolipids, and glycerolipids. The depletion of bis(monoacylglycerol)phosphate (BMP) species suggests perturbations in endolysosomal lipid homeostasis and membrane trafficking pathways. Increased levels of ether-linked lysophosphatidylcholine (LPC-O) species, together with altered phosphatidylethanolamine (PE) and phosphatidylserine (PS) species, indicate extensive membrane lipid remodeling and changes in cellular signaling. Furthermore, the accumulation of diacylglycerol (DG) and triacylglycerol (TG) species points to disturbances in lipid storage and metabolism. Overall, these findings indicate that aromatic OPFRs induce cytotoxicity, oxidative stress, and alteration of cholinergic function, and are associated with lipid dysregulation linked to neurotoxicity in brain organoids. Future research should explore chronic low-dose exposure and long-term neurological effects.

## 1. Introduction

Organophosphate flame retardants (OPFRs) have become common additives in consumer products, replacing polybrominated diphenyl ethers (PBDEs) due to restrictions on their use, driven by concerns about environmental persistence and toxicity [1]. OPFRs are present in furniture, electronics, textiles, and children’s products and are often not chemically bound to these materials, which facilitates their release into the environment through volatilization, abrasion, or leaching [2,3,4]. Therefore, human exposure is widespread and primarily occurs via inhalation, ingestion, and skin contact [5,6,7,8].

Although OPFRs were initially considered safer alternatives to PBDEs, evidence has challenged this assumption in consideration of their effects on the nervous system [9]. Neurotoxicity is one of the most concerning endpoints associated with OPFR exposure, since these compounds and their metabolites can cross the blood–brain barrier and bioaccumulate in brain tissue [10]. The neurotoxic effects of OPFRs are linked to their chemical structures, which determine how these compounds are absorbed and distributed throughout the body [11,12]. For instance, chlorinated OPFRs such as tris(1,3-dichloro-2-propyl) phosphate (TDCPP), tris(2-chloroisopropyl) phosphate (TCIPP), and tris(2-chloroethyl) phosphate (TCEP) often bioaccumulate due to their reduced metabolic rates and longer half-lives [13], whereas aryl OPFRs such as triphenyl phosphate (TPhP) with aromatic rings exhibit stronger interactions with biological receptors and greater disruptive potential [14].

Neuronal cell models have become essential tools for investigating how they cause toxicity in affected environments. Specifically, the human neuroblastoma SH-SY5Y cell line is a widely used in vitro cell model for neurotoxicology in human research [15]. SH-SY5Y cells are a reliable and consistent model for exploring key neurotoxic mechanisms, including oxidative stress, mitochondrial dysfunction, and alterations in neurotransmission pathways. Their human origin, experimental stability, and responsiveness to chemical stressors make them ideal for screening neurotoxic effects and conducting mechanistic studies of environmental contaminants [15]. This cell line has been used to assess the neurotoxicity of flame retardants, including TDCPP, TPhP, and other brominated compounds [16]. At the tested concentrations, none significantly modulated cell viability, neural plasticity or development, mitochondrial function, or oxidative stress and inflammation.

The use of three-dimensional cell culture models in neurotoxicity studies can more accurately reflect the complex effects of OPFRs on the nervous system. For instance, a study using 3D rat brain organotypic cultures and human-derived neural spheres confirmed that even low micromolar exposures to TPhP can reduce neurotransmitter levels, including gamma-aminobutyric acid (GABA) and glutamate, induce astrogliosis, and alter pathways related to synaptic signaling and action potential transmission [9]. Other sophisticated cellular models to study neurotoxicity are brain organoids. These structures are 3D neural models derived from human pluripotent stem cells that recapitulate key aspects of early brain development. Containing multiple neural cell types such as neurons, oligodendrocytes, and glial cells, they better model neural differentiation, synaptic connectivity, and cell–cell communication than conventional 2D neuronal cultures [17,18]. Consequently, brain organoids provide a physiologically relevant human-based platform for investigating neurodevelopmental processes and chemical-induced neurotoxicity [19].

Omics technologies, such as transcriptomics, proteomics, metabolomics and lipidomics, enable molecular-level analysis of biological systems. Specifically, lipidomics focuses on the comprehensive profiling of lipid species, which play essential roles not only in membrane structure but also in energy metabolism and cellular signaling. Since lipid composition is closely linked to neuronal function, changes in lipid profiles can reveal disruptions in pathways that regulate membrane integrity, oxidative stress responses, and neurotoxic signaling [20]. Consequently, lipidomics provides valuable insight into cellular responses to external stimuli and environmental stressors [21]. For example, disruptions in brain lipid homeostasis are linked to impaired neuronal membrane integrity, altered neurotransmission, and neuroinflammation, processes that can ultimately lead to damage to the central nervous system and neurodegeneration.

The OPFRs examined in this study were selected due to their high prevalence in household dust, as previously reported [5]. The present study aims to investigate the neurotoxic potential of OPFRs by assessing phenotypic alterations in SH-SY5Y cells and brain organoids, alongside lipidomic profiling of brain organoids, thereby advancing our understanding of the mechanisms underlying OPFR-induced neurotoxicity.

## 2. Materials and Methods

### 2.1. Chemicals and Reagents

The OPFRs TDCPP, TPhP, TCEP, TCP, and EHDPhP were purchased from Sigma-Aldrich (Darmstadt, Germany), while tris(2-butoxyethyl) phosphate (TBOEP) and tris (2-ethylhexyl) phosphate (TEHP) were obtained by Dr. Ehrenstorfer GmbH (Augsburg, Germany). The OPFR stock solutions were prepared in dimethyl sulfoxide (DMSO) at 100 mM. The lipid standards used were C12:0 ceramide, C12:0 glucosylceramide, 1,2,3-17:0 triacylglycerol, 17:1 lysophosphatidylethanolamine, and 16:0 D31–18:1 phosphatidylethanolamine, all sourced from Avanti Polar Lipids (Alabaster, AL, USA), and mixed in methanol. High-purity DMSO (99.9%), formic acid (99.9%), 2′,7′-dichlorofluorescein diacetate (DCFDA), and phosphate-buffered saline (PBS) were purchased from Merck (Darmstadt, Germany). Chloroform (99.9% purity) was acquired from Carlo Erba Reagents (Sabadell, Spain). Chromatographic mobile phases were prepared with LC-MS-grade methanol and water from Fisher Chemicals (Waltham, MA, USA). Ultrapure water was produced using a Milli-Q Integral 3 water purification system (Merck, Darmstadt, Germany).

### 2.2. Cell Culture

The SH-SY5Y cell line was obtained from the American Type Culture Collection (ATCC, Manassas, VA, USA; CRL-2266). The culture medium used was Ham’s F-12K Nutrient Mixture, Kaighn’s Modified (Corning, NY, USA), supplemented with 10% fetal bovine serum (10270-106, Gibco, Thermo Fisher Scientific, Waltham, MA, USA). Cells were maintained in vented 75 cm^2^ polystyrene flasks and incubated at 37 °C in a humidified 5% CO_2_ atmosphere.

Neurocortical cerebral organoids were generated from the human induced pluripotent stem cell line BJiPSC-SV4F-9 following an established protocol [22]. Organoid formation and maturation were carried out over a 40-day differentiation process using defined spheroid culture conditions and staged supplementation with growth and differentiation factors. Briefly, iPSCs were first expanded on Geltrex-coated culture surfaces and subsequently seeded into low-adhesion U-bottom 96-well plates to promote spheroid aggregation. At this stage, cells were maintained in spheroid induction medium containing selected inhibitors and growth factors to support early spheroid formation. Over subsequent culture stages, the medium composition was progressively adjusted, incorporating factors such as basic fibroblast growth factor (bFGF) and epidermal growth factor (EGF) to enhance spheroid growth and stability. At day 20 of differentiation, developing spheroids were transferred to ultra-low-attachment 96-well plates for continued culture. Neurocortical differentiation was initiated during the final phase of the protocol, between days 27 and 40, by switching to a neurobasal-based medium supplemented with brain-derived neurotrophic factor (BDNF) and neurotrophin-3 (NT-3).

SH-SY5Y cells were selected for the initial evaluation of OPFR-induced cellular responses due to their robustness and reproducibility as a two-dimensional neuronal model, while lipidomic analyses were performed in brain organoids to observe OPFR-related changes in a more complex, physiologically relevant three-dimensional neural environment.

### 2.3. Biological Assays

Cell viability. The SH-SY5Y cells were seeded into flat-bottomed 96-well microplates (Nunc, ThermoFisher Scientific, Waltham, MA, USA) at a density of 10^5^ cells/mL. After 24 h, attached cells were exposed to 0.5, 0.25, and 0.125 μL of 20 mM OPFR stock solutions in DMSO, in a final volume of 100 μL of complete medium. The final tested concentrations were 100, 50, 25, and 12.5 μM, respectively. Control wells were prepared using DMSO as a vehicle (0.5, 0.25, and 0.125% (*v*/*v*), respectively). All conditions were performed in triplicate. After 72 h of incubation under standard conditions, cell viability was measured using the resazurin assay with CellTiter-Blue^®^ reagent (Promega Corporation, Madison, WI, USA), following the manufacturer’s instructions. The fluorescent signal was measured after 4 h of incubation (excitation/emission wavelengths of 560/590 nm) using a microplate fluorescence reader (Infinite M Plex, Tecan, Männedorf, Switzerland).

Reactive Oxygen Species (ROS) assay. SH-SY5Y neuroblastoma cells were plated in 96-well plates at a final density of 1 × 10^5^ cells/mL. After 24 h of culture, cells were rinsed with PBS and loaded with the fluorescent probe by incubation with 100 μL of PBS containing 11 mM glucose and 20 μM DCFDA for 30 min. Following incubation, the medium containing the dye was aspirated, and cells were washed twice using PBS. Cells were then exposed to complete culture medium containing the OPFRs at a final concentration of 100 μM. DMSO was used as a vehicle control. All experimental conditions were performed in triplicate. Intracellular fluorescence was monitored at 15 min intervals over a 2 h period using a fluorescence microplate reader (Infinite M Plex, Tecan, Männedorf, Switzerland) maintained at 37 °C, with excitation and emission wavelengths set to 480 and 520 nm, respectively. The 2 h endpoint was selected to assess the ROS formation before the activation of cellular antioxidant and adaptive mechanisms. The high concentration was chosen to maximize ROS detection while limiting the impact of antioxidant defense mechanisms.

Acetylcholinesterase (AChE) Inhibition Assay. AChE inhibition was evaluated using a colorimetric assay based on the Ellman method [23]. SH-SY5Y cells were seeded at 10^5^ cells/mL into a 96-well microplate. After 24 h incubation, the medium was removed, and the wells were washed twice with PBS. For organoid experiments, each sample consisted of a single organoid cultured in an individual well of a round-bottom 96-well plate. Before the assay, the culture medium was aspirated, and the organoids were washed with PBS. Cells and organoids were then exposed to 100 μL of complete culture medium containing OPFRs at a final concentration of 100 μM, prepared by adding 0.5 μL of a 100 mM OPFR stock solution. Control wells received 0.5 μL of DMSO as the vehicle control. The organophosphorate pesticide chlorpyrifos at 100 μM was used as a positive control of AChE inhibition to validate the assay. This high concentration was employed to assess inhibitory potential while enhancing the sensitivity for detecting enzymatic modulation. Each experimental condition was performed in triplicate. After 3 h of exposure, the medium was removed and the AChE activity was determined by adding to each well 100 µL of a mixture containing 8 mM acetylthiocholine iodide (ATCI) as the enzymatic substrate and 10 mM 5,5′-dithiobis(2-nitrobenzoic acid) (DTNB) in Opti-MEM medium (Gibco, Thermo Fisher Scientific, Waltham, MA, USA). The hydrolysis of ATCI by AChE releases thiocholine, which reacts with DTNB to form a yellow-colored product. The change in absorbance was measured spectrophotometrically every minute for 30 min at 410 nm using a microplate reader (Infinite M Plex, Tecan, Männedorf, Switzerland). The percentage of AchE activity relative to the vehicle condition was calculated from the slopes of yellow-colored product formation over 30 min.

### 2.4. Lipid Extraction and Sample Preparation from Brain Organoids Exposed to OPFRs

Lipidomics studies were performed using 3D brain organoid cultures. In a 96-well plate, each well held a brain organoid produced as described above, with six wells per OPFR [22]. For the exposure experiments, the culture medium was gently removed and replaced with 100 μL of fresh Neurobasal medium supplemented with OPFRs or vehicle. Appropriate volumes of OPFR stock solutions (20 mM and 2 mM for high and low doses, respectively) or DMSO were added to achieve final concentrations of 25 μM and 2.5 μM. These concentrations were selected based on viability data confirming the absence of cytotoxic effects, allowing lipidomic alterations to be assessed independently of cell death–associated responses. Following a 72 h exposure period, the medium was discarded, and organoids were rinsed twice with PBS, then stored frozen until lipid extraction.

Lipid extraction was initiated by adding 100 μL of ultrapure water to each organoid and transferring the samples to glass vials. Methanol (250 μL) and chloroform (500 μL) were then added sequentially. Before sample homogenization, 5 μL of an internal standard mixture containing 200 pmol of each lipid class were added. After vortexing and sonication for 15 min, the extracts were transferred to Eppendorf tubes, and a nitrogen stream was used to evaporate the solvents. The dried lipid residues were reconstituted in 180 μL of methanol, followed by centrifugation at 12,000× *g* for 10 min. Then, 130 μL of the supernatant were finally transferred to glass conical MS vials for analysis. Quality control samples consisted of a pool of 20 μL of the supernatant of each extract in separate glass vials. Prepared vials were stored at −80 °C until analysis.

### 2.5. UHPLC-HRMS Lipidomics and Data Processing

Lipidomic profiling was performed using an ultra-high-performance liquid chromatography system (ACQUITY UPLC), coupled to a SELECT SERIES Cyclic ion mobility mass spectrometer, both from Waters Corporation (Milford, MA, USA). Samples and quality-control pools were analyzed in a randomized acquisition sequence. Aliquots of 5 μL were injected onto a reversed-phase Kinetex C8 column (100 × 2.1 mm, 1.7 μm particle size; Phenomenex, Torrance, CA, USA) maintained at 30 °C. Chromatographic separation was achieved at a flow rate of 0.3 mL/min using water supplemented with 2 mM ammonium formate and 0.2% formic acid as mobile phase A, and methanol containing 1 mM ammonium formate and 0.2% formic acid as mobile phase B. The gradient elution began at 20% phase A, followed by a linear decrease to 10% A over 3 min and an isocratic step until 6 min. Phase A was then reduced to 1% over the next 9 min and maintained for 3 min before returning to the initial conditions, resulting in a total run time of 20 min.

Mass spectrometric analyses were performed in high-definition MS (HDMS) mode using cyclic ion mobility-enabled MS/MS acquisition. Instrument parameters were set as follows: capillary voltage 3.0 kV, cone voltage 10 V, source temperature 120 °C, desolvation temperature 450 °C, desolvation gas flow 800 L/h, and nebulizer pressure 6.0 bar. The reference capillary was operated at 3.0 kV, with an infusion flow of 10 μL/min. Collision energies were set to 10 V in the trap cell and 4 V in the transfer region, with a transfer RF voltage of 200 V. Data were acquired across an m/z range of 50–1200 for both MS and MS/MS using a scan time of 0.55 s. Ion mobility separation was performed using a single pass under default conditions (entrance voltage 5 V, array offset 2 V, racetrack bias 10 V, travelling-wave velocity 375 m/s, and wave height 6 V).

Raw LC-MS data were processed with Progenesis QI software v31.49.1234 (Nonlinear Dynamics, Waters, Milford, MA, USA) following an untargeted lipidomics workflow. The MS-DIAL MS/MS lipid spectral library was used to perform lipid identification within the same platform. Only features achieving identification confidence scores above 50%, based on combined MS and MS/MS matching criteria, were considered reliable for downstream analyses.

### 2.6. Statistical Analysis

The multivariate statistical analysis of lipidomic data was performed using PCA to identify key multivariate response patterns associated with OPFR treatment groups and to quantify each pattern’s contribution to the total explained variance. Partial least squares discriminant analysis (PLS-DA) was employed to develop supervised models that differentiate samples treated with each OPFR from those exposed to the vehicle control [24,25]. Data was autoscaled, and leave-one-out was the cross-validation method for model evaluation. Classification performance was assessed using sensitivity, specificity, class error, and the Matthews Correlation Coefficient (MCC). MCC values range from −1 to 1, with values closer to 1 indicating stronger predictive discrimination, values near 0 indicating random classification, and negative values indicating systematic misclassification [24]. Lipid species contributing to class discrimination were selected using two criteria: a Variable Importance in Projection (VIP) score greater than 1 in the PLS-DA model and a *p*-value < 0.05 from univariate comparisons between treated samples and controls using a *t*-test with multiple hypothesis testing (MHT) correction. This workflow, including PCA, supervised PLS-DA modeling, and lipid selection based on multivariate (VIP scores) and univariate (*p*-values) criteria, identified the lipid species associated with each OPFR exposure.

### 2.7. Computational Environment and Software

A Fujitsu Celsius R940n workstation with dual Intel Xeon E5-2620v3 processors and 256 GB of RAM, running Microsoft Windows 10 was employed for all the computations. The analyses were conducted using MATLAB R2025a (The MathWorks, Inc., Natick, MA, USA), including the Bioinformatics Toolbox (version R2025a). The PLS-Toolbox (Eigenvector Research Inc., Manson, WA, USA) was employed to perform Principal Component Analysis (PCA) and Partial Least Squares Discriminant Analysis (PLS-DA). Graphical abstract was created using BioRender.com (BioRender, Toronto, ON, Canada).

## 3. Results

### 3.1. Cell Viability, Induction of Reactive Oxygen Species (ROS), and Acetylcholinesterase (AChE) Activity

**Figure 1:**
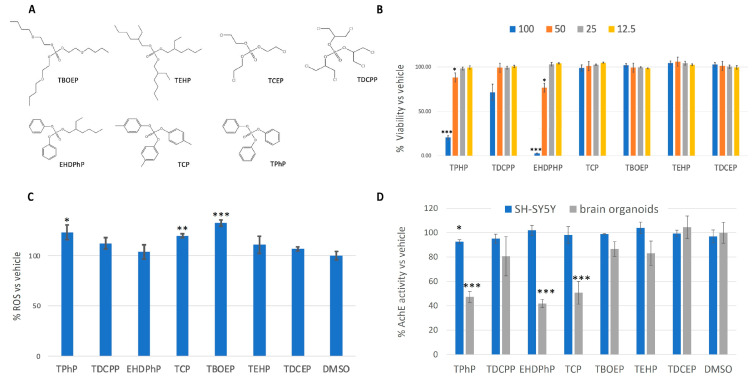
Chemical structure of OPFRs and biological effects on SH-SY5Y cells. Structures of the OPFRs employed (**A**); cell viability (**B**), ROS generation (**C**), and AChE activity (**D**), expressed as percentages relative to vehicle samples incubated DMSO. Significant differences relative to cells treated with the vehicle are shown: * *p*-value < 0.05, ** *p*-value < 0.01, *** *p*-value < 0.005. Experiments were performed in triplicate; error bars show standard deviation. TPhP: triphenylphosphate; TDCPP: tris(1,3-dichloro-2-propyl) phosphate; EHDPhP: 2-ethylhexyldiphenyl phosphate; TCP: tricresyl phosphate; TBOEP: tris(2-butoxyethyl) phosphate; TEHP: tris (2-ethylhexyl) phosphate; TDCEP: tris(2-chloroethyl) phosphate; DMSO: dimethyl sulfoxide (vehicle).

First, the biological effects of the selected OPFRs (Figure 1A) on SH-SY5Y cells were evaluated by assessing cell viability, ROS production, and AChE inhibition.

The cell viability assays of the seven OPFRs examined at concentrations of 12.5, 25, 50, and 100 µM revealed that only EHDPhP and TPhP significantly induced cell death at 100 µM, reducing cell viability to 3% and 21% relative to the vehicle control, respectively. Interestingly, both compounds contain aromatic rings in their chemical structure. TDCPP exposure caused a discrete decrease in cell viability (to 72%), although this effect was not statistically significant. The viability of SH-SY5Y cells was not disturbed at lower doses (Figure 1B).

ROS production and AChE activity were assessed at 100 µM, the concentration at which lethal effects were observed. After 2 h of exposure, TBOEP, TPhP, and TCP induced significant increases in ROS levels (133%, 123%, and 120%, respectively), whereas the other OPFRs also showed a trend toward increased ROS production, although these changes were not statistically significant (Figure 1C). Interestingly, despite the marked cytotoxicity induced by EHDPhP and TPhP, ROS levels were comparable to those elicited by other non-cytotoxic OPFRs, such as TBOEP and TEHP, suggesting that cell death mechanisms other than excessive oxidative stress may contribute to the toxicity of these compounds.

The evaluation of AChE activity revealed that only TPhP produced a small but statistically significant reduction after 3 h of exposure, decreasing enzyme activity to 93% relative to the DMSO control. No significant effects on AChE activity were observed for the other OPFRs (Figure 1D). Interestingly, when AChE activity was evaluated in 40-day-old neurocortical organoids derived from the human BJiPSC-SV4F-9 cell line under the same exposure conditions, significant inhibition was observed for TPhP, EHDPhP, and TCP, reducing enzyme activity to 47%, 42%, and 51% of the vehicle control, respectively. These findings indicate a stronger impact of these compounds on cholinergic function in the organoid model and suggest that more physiologically relevant and structurally complex neuronal systems may be more sensitive to OPFR-induced neurotoxic effects than SH-SY5Y cells.

Overall, these results indicate that OPFRs bearing aromatic rings, specifically EHDPhP, TPhP and TCP (Figure 1A), elicited more pronounced alterations in the cellular phenotype compared to non-aromatic OPFRs across the endpoints evaluated. Based on this differential toxicity profile, a more in-depth investigation of lipidomic alterations in brain organoids was subsequently conducted, focusing exclusively on OPFRs containing aromatic rings. Accordingly, lipidomic analyses were performed using the three aryl OPFRs included in this study—TPhP, EHDPhP, and TCP—to explore the role of the aromatic-dependent effects of OPFRs on brain lipid homeostasis.

### 3.2. Lipidomics of Brain Organoids Exposed to OPFRs

**Figure 2:**
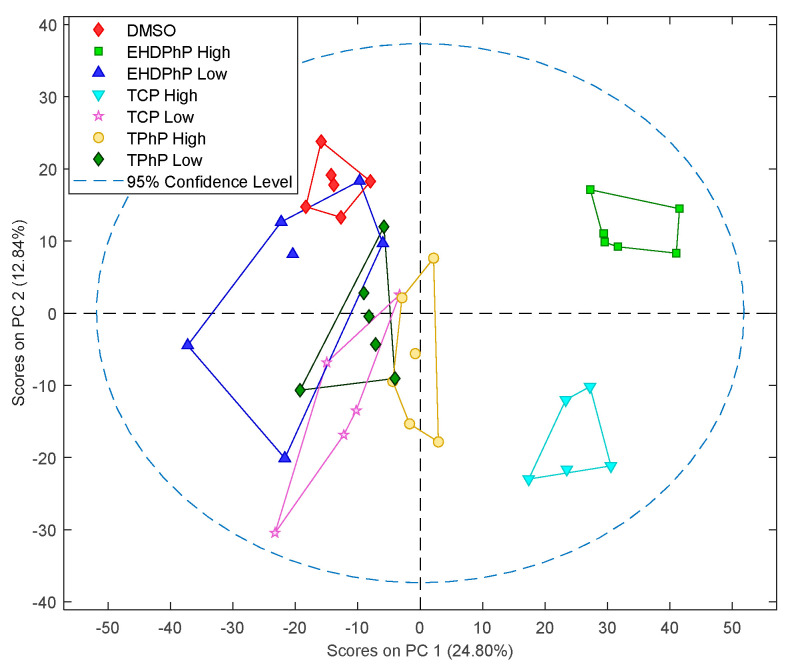
Principal component analysis (PCA) of lipidomic response profiles in brain organoids exposed to TCP, EHDPhP, and TPhP at low and high concentrations (2.5 and 25 μM).

To investigate the effects of the aromatic OPFRs on brain-like systems, 40-day-old neurocortical organoids were exposed to two concentrations (2.5 and 25 μM; hereafter referred to as low and high dose) of the three aryl OPFRs: TCP, EHDPhP, and TPhP. After 72 h of exposure, no cell death was observed at the concentrations tested. Lipid extracts were subjected to untargeted lipidomic profiling. An initial exploratory overview of the dataset was obtained through PCA. As shown in Figure 2, PC1 (explaining 25% of the variance) clearly distinguished the high-dose treatments of TCP and EHDPhP from vehicle and low-dose samples, indicating a common lipidomic response at the highest concentrations. TPhP high-dose samples were also shifted along PC1, though less markedly than TCP or EHDPhP. PC2 (explaining 13% of the variance) further separated high-dose TCP from high-dose EHDPhP, suggesting partially distinct lipid perturbations between these two compounds.

**Figure 3:**
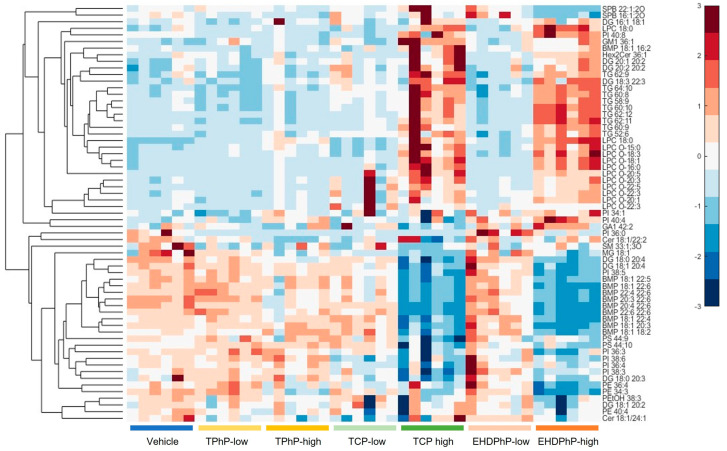
Hierarchical clustering heatmap of significantly altered lipid species in brain organoids exposed to TCP, EHDPhP, and TPhP at 2.5 and 25 µM (low and high dose). Lipids were selected based on VIP scores > 1 and *p*-values < 0.05 in at least one comparison of an OPFR treatment versus the vehicle. Abbreviations: BMP, bis(monoacylglycerol)phosphate; Cer, ceramide; DG, diacylglycerol; GA1, asialo-ganglioside GM1; Hex2Cer, hexosylceramide; LPC, lysophosphatidylcholine; PE, phosphatidylethanolamine; PI, phosphatidylinositol; PS, phosphatidylserine; SM, sphingomyelin; SPB, sphingoid base; TG, triacylglycerol.

To refine these observations, separate PLS-DA models comparing each OPFR vs. vehicle were generated. In all cases, exposure at 25 μM was clearly distinguished from the vehicle group, showing high classification accuracy (MCC = 1). Lipid species with VIP scores > 1 and *p*-values < 0.05 in at least one of the comparisons were selected and combined for further analysis. These lipids were then subjected to hierarchical clustering to identify common patterns of lipid changes (Figure 3). The heatmap revealed distinctive clusters at the highest doses of TCP and EHDPhP, particularly within glycerolipids and glycerophospholipids.

**Figure 4:**
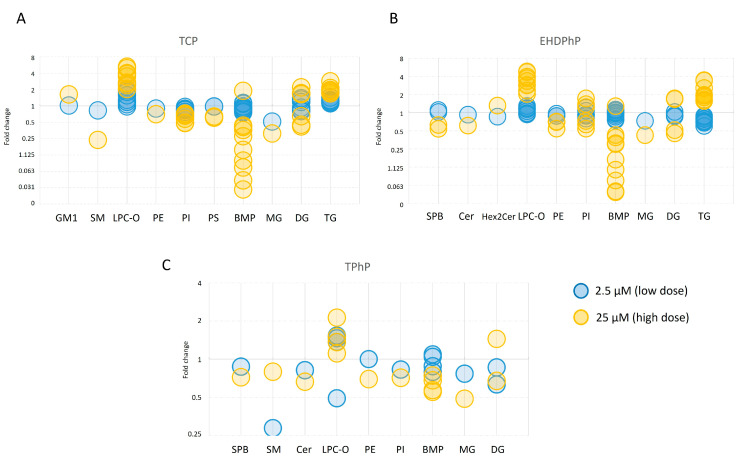
Bubble plots illustrating fold changes in lipid classes in brain organoids exposed to (**A**) TCP, (**B**) EHDPhP, and (**C**) TPhP at low (2.5 μM, blue) and high (25 μM, yellow) concentrations. Each point represents an individual lipid species within a specific class. Fold change values > 1 indicate increased abundance, while values < 1 indicate decreased abundance compared to the vehicle control. Abbreviations: BMP, bis(monoacylglycerol)phosphate; Cer, ceramide; DG, diacylglycerol; GM1, ganglioside GM1; Hex2Cer, dihexosylceramide; LPC-O, ether lysophosphatidylcholine; MG, monoacylglycerol; PE, phosphatidylethanolamine; PI, phosphatidylinositol; PS, phosphatidylserine; SM, sphingomyelin; SPB, sphingoid base; TG, triacylglycerol.

Comparison of lipid changes revealed several common effects across TCP, EHDPhP, and TPhP treatments. For a more detailed view of the lipidomic changes caused by each OPFR, bubble plots showing fold changes in various lipid classes across different concentrations are shown in Figure 4. Additional information on individual lipid species, their abundances, and their identification is provided in Supplementary Table S1. Among glycerophospholipids, the most pronounced change was a dose-dependent decrease in bis(monoacylglycerol) phosphate (BMP) species. This decrease was observed across all OPFRs; however, it was more substantial and affected a broader range of BMP species for TCP and EHDPhP. The most strongly affected BMP species contained acyl chains of 18:1 and 22:6, the latter corresponding to the structure of docosahexaenoic acid (DHA), the most abundant polyunsaturated fatty acid in the brain. Whereas the decrease at the high dose of TPhP ranged from 0.9- to 0.5-fold, reductions observed for EHDPhP and TCP ranged from 0.4- to 0.05-fold, indicating a near-complete depletion of certain BMP species.

BMPs are highly enriched in intraluminal membranes of late endosomes and lysosomes, where they play a role in lipid degradation, membrane dynamics and vesicular sorting. A decrease in BMP levels could reflect alterations in endolysosomal homeostasis, potentially affecting lipid trafficking and regulation of lipid metabolism. Another key change was the rise in ether-linked lysophosphatidylcholine (LPC-O) species, consistently observed at high doses across all OPFRs. The dose–response was again strongest for TCP and EHDPhP, with fold-changes ranging from 2- to 16-fold. The concomitant increase in LPC-O species and depletion of BMPs points to substantial remodeling of intracellular membrane lipids, suggesting perturbations in the endolysosomal system rather than a simple impairment of degradative activity. To further explore lysosomal involvement, lipid degradation indices were estimated using the ratios of sphingomyelin (SM) to sphingosine, phosphatidylcholine (PC) to lysophosphatidylcholine (LPC), and ether-linked phosphatidylcholine (PC-O) to LPC-O (Table S2). These indices were markedly elevated following exposure to high concentrations of EHDPhP, TCP, and TPhP, indicating enhanced membrane lipid turnover and catabolic processing. This finding supports the notion that aromatic OPFRs induce a profound disruption of endolysosomal lipid homeostasis, characterized by altered lipid trafficking and membrane remodeling.

In addition, glycerophospholipids showing significant, dose-dependent decreases under EHDPhP and TCP exposure included several phosphatidylethanolamine (PE) and phosphatidylinositol (PI) species. These decreases, though variable in size (fold changes detailed in Table S1), suggest changes in membrane composition, curvature regulation and signaling pathways, given PE and PI’s central roles in membrane dynamics and lipid signaling [26].

Regarding glycerolipids, we observed a significant increase (from 1.5 to 3.5-fold) in polyunsaturated long-chain diacylglycerol (DG) and triacylglycerol (TG) species, including DGs with 40 carbons and 3 to 6 unsaturations, and TGs ranging from 58 to 64 carbons with 8 to 12 unsaturations, at the highest doses of TCP and EHDPhP. Conversely, DG species with 38 carbons were reduced by half. This accumulation could be related to the previously mentioned altered degradative activity, which breaks down membrane phospholipids and releases free fatty acids. These fatty acids could then be re-esterified onto the glycerol backbone, forming DG and TG as part of a detoxification and lipid-storage response. Consistent with this, monoacylglycerol (MG) levels decreased under the same conditions, suggesting a shift toward DG/TG synthesis and altered lipid metabolism.

## 4. Discussion

This study demonstrates that exposure to OPFRs induces phenotypic and lipidomic changes in human neural models. Using SH-SY5Y cells and 3D brain organoids, the study showed that OPFRs disrupt cell viability, oxidative balance, cholinergic function, and lipid metabolism. Among the seven OPFRs examined, the aromatic rings-bearing compounds TPhP, EHDPhP, and TCP produced the most pronounced effects, suggesting a potential structure–toxicity relationship. In phenotypic assays, TPhP and EHDPhP showed the most significant cytotoxicity, substantially reducing cell viability. Regarding ROS, all OPFRs tested induced ROS generation, with TPhP, TCP and TBOEP inducing the highest responses. The antioxidant mechanisms of cells can be overwhelmed by ROS, leading to oxidative stress; which in turn provokes oxidation of lipids, proteins, and DNA, causing cellular damage and death. These increases likely drive the observed phenotypic changes and may be early factors in the lipidomic alterations observed in this study [25].

A significant body of research on OPFR neurotoxicity has used animal models, including rodents, zebrafish, and also in vitro models such as 2D neuronal cultures [27]. These studies have shown that OPFRs such as TPhP, TDCPP, and TCEP can induce oxidative stress, cell death, and disruption of neurodevelopmental signaling pathways [12,16]. However, classical non-animal models do not fully reflect the structural and cellular complexity of the human central nervous system (CNS) [16]. In this context, human brain organoids are an important methodological advance because they can more accurately reproduce complex neurodevelopmental responses to toxicants and provide better translational relevance [28]. The current study used this model to assess OPFR-induced inhibition of AChE and to explore lipid changes under aromatic OPFR exposure, making the findings more applicable to human neurotoxicity. Interestingly, a marked inhibition of AChE activity was observed only for the aromatic OPFRs TPhP, EHDPhP, and TCP in neurocortical organoids, whereas SH-SY5Y cells showed only a modest reduction following TPhP exposure. These findings suggest that the cholinergic effects of OPFRs are highly model-dependent and may be more readily detected in physiologically relevant systems that better recapitulate the cellular complexity of the human brain.

BMP is enriched in lysosomal membranes and plays a vital role in lipid trafficking and degradation [29]. Reduced BMP levels are linked to impaired lysosomal function and are characteristic of lysosomal storage disorders and neurodegenerative diseases such as Batten disease [30,31] and Alzheimer’s disease [32]. The observed decrease in BMP species after exposure to TPhP, EHDPhP, and TCP suggests altered endosomal–lysosomal lipid processing [33]. This alteration may affect lipid processing and intracellular lipid distribution, contributing to broader disturbances in cellular lipid metabolism.

The lipidomic profile also revealed a marked increase in LPC-O species following exposure to TCP, EHDPhP, and TPhP. Together with the depletion of BMPs, this finding points to substantial remodeling of intracellular membrane lipids and disruption of endolysosomal homeostasis. As LPC-O species originate from ether-linked phosphatidylcholines (plasmalogens), their accumulation may indicate enhanced degradation of neuronal membrane phospholipids. Consistent with this hypothesis, alterations in the lipid degradation indices of SM, PC and PC-O, further suggest changes in lipid catabolism and trafficking. Decreases in PI and PE were also observed across all three OPFRs, whereas lower PS levels were observed after TCP exposure. Because these phospholipids play essential roles in membrane structure, signaling, trafficking, and apoptotic regulation, their depletion may contribute to impaired neuronal function [26].

Increases in DG and TG species indicate a cellular response to lipid imbalance. As a protective mechanism to mitigate lipotoxicity, fatty acids released from BMP and other hydrolyzed phospholipids may be redirected toward neutral lipid storage pools, such as TGs [34]. Consistent with this interpretation, the TG species that increased in the present study for TCP and EHDPhP were characterized by a high total carbon number and polyunsaturated acyl chains, matching those of the BMP species that were decreased. This detoxification-like response is often observed during oxidative stress and membrane disruption [35]. However, the sustained accumulation of these storage lipids could lead to metabolic dysregulation and has been associated with neurodegenerative diseases [31,36].

Furthermore, a specific decline in GM1 ganglioside was observed with TCP exposure. Gangliosides are vital for neuronal development, synaptic function, and signal transduction [37], and their decrease may impair normal brain functioning. This observation is consistent with previous studies indicating that aryl OPFRs disrupt synaptic and membrane-related pathways [9].

In general, the lipid changes observed for TCP and EHDPhP are very similar, and some of the changes observed for these compounds were also present in TPhP-exposed cells, but with less intensity. The closer resemblance between TCP and EHDPhP, compared with TPhP, suggests a structure–activity relationship in which molecular symmetry and aromatic substitution influence membrane interactions and downstream lipidomic effects. Compared with TPhP, TCP and EHDPhP exhibit lower water solubility and higher log Kow values (Table S3), indicating a greater potential for bioaccumulation that may be associated with the more pronounced lipid alterations observed. In addition, TCP and EHDPhP display slower metabolic rates than the other OPFRs tested (with the exception of TEHP), suggesting increased persistence within the brain organoid system. This prolonged retention may enhance their interactions with lipid compartments and cellular membranes, thereby contributing to the greater lipid dysregulation detected for these compounds in the present study. Considering these findings, together with the higher levels of cell death induced by EHDPhP, this compound may represent the OPFR with the highest neurotoxic potential among those evaluated. TCP could be considered the second most impactful compound, as it induced elevated ROS levels and marked lipid alterations. Although TPhP also induced substantial cell death and ROS generation, its higher metabolic turnover may limit the duration of its interaction with cellular targets, potentially explaining the comparatively milder effects observed in the lipidomics analysis.

Overall, the observed lipid alterations, including BMP depletion and accumulation of storage lipids, suggest the involvement of pathways related to endolysosomal homeostasis and neurodegeneration. These lipid signatures are consistent with alterations previously reported in neurodegenerative disorders such as Batten disease [30] and Alzheimer’s disease [32]. However, additional functional studies are required to determine whether these lipid changes result in altered lysosomal function and contribute to neurodegenerative mechanisms.

Evidence from previous studies indicates that OPFRs can reach the central nervous system by crossing the blood–brain barrier (BBB). In particular, aromatic OPFRs such as TPhP have been shown to penetrate the BBB, leading to vascular dysfunction and increased BBB permeability in brain tissue [38]. Experimental evidence further demonstrates that TPhP and its metabolite diphenyl phosphate (DPP) induce transcriptomic and metabolomic changes associated with neurotoxicity [39]. Generally, organophosphate compounds are known to compromise BBB structure and function, thereby facilitating the entry of neurotoxic substances into the brain [40]. In line with this, given the central role of AChE in regulating cholinergic signaling, the observed inhibition suggests that cholinergic pathways may represent a relevant target of aromatic OPFR neurotoxicity. However, further studies are required to determine whether the observed effects result from direct interaction with the enzyme or from indirect alterations in cellular metabolism and neuronal homeostasis.

Together, these findings reinforce the biological plausibility of direct OPFR effects on brain tissue and highlight the relevance of human brain organoids as physiological models to study OPFR-induced neurotoxicity.

Despite these findings, this study has several limitations. First, it focuses on acute exposure conditions and employs relatively high OPFR concentrations, which may not fully reflect the complexity of real-world environmental exposure scenarios. Additionally, the exclusive use of lipidomics to characterize OPFR-induced effects restricts the interpretation of the results. Future studies should therefore investigate the effects of chronic, low-dose exposure to aromatic OPFRs, particularly in relation to lipid dysregulation and pathways associated with cognitive impairment and neurodegenerative diseases. The use of more physiologically relevant models, such as brain organoids, combined with multi-omics approaches and functional analyses, will be essential to improve neurotoxicological risk assessment and to better elucidate the long-term health consequences of OPFR exposure.

## 5. Conclusions

The present study demonstrates that exposure to OPFRs induces neurotoxic effects in human neural cell models, affecting both cell phenotype and lipid composition. Among the seven OPFRs evaluated, the aromatic compounds TPhP, EHDPhP, and TCP showed the most significant responses, suggesting that their aromatic structures and high lipophilicity may increase toxicity and thereby disrupt neural cell homeostasis. By integrating SH-SY5Y neuroblastoma cells for phenotypic screening and neurocortical brain organoids for lipidomic profiling, this work enabled us to better understand the cytotoxic mechanisms of OPFRs in neural cells. Lipidomic analysis showed consistent disruptions mainly in phospholipid, and glycerolipid classes, indicating compromised endolysosomal function and phospholipid composition remodeling. The accumulation of DG and TG species further supports adaptive responses to oxidative stress and membrane damage, reflecting a shift toward lipid storage and detoxification mechanisms. Based on the combined evidence from lipidomics, cell viability, ROS generation, and AChE inhibition, EHDPhP showed the strongest overall effects, followed by TCP and TPhP. Overall, the findings indicate that exposure to aromatic OPFRs may trigger oxidative stress and profound lipid remodeling associated with lysosomal dysfunction, potentially contributing to neurodevelopmental alterations and neurodegenerative processes. Although this study has several limitations, including the use of acute exposure conditions and relatively high OPFR concentrations, the findings raise concerns about the safety of widely used OPFRs. These results highlight the need for further research under chronic, low-dose exposure conditions that more accurately reflect real-world scenarios. Future studies incorporating advanced human-relevant models and multi-omics approaches will be essential to improve risk assessment and to achieve a more comprehensive understanding of the long-term neurological effects of OPFR exposure.

## Data Availability

The original contributions presented in this study are included in the article/Supplementary Material. Further inquiries can be directed to the corresponding authors.

## Supplementary Materials

The following supporting information can be downloaded at https://www.mdpi.com/article/10.3390/toxics14070555/s1: Table S1. List of significant lipid changes across treatments in brain organoids. Table S2. Degradation indices of lipids processed in the lysosome. Table S3. Physicochemical properties and metabolic transformations of the OPFRs studied.
